# Oral and Gut Microbial Diversity and Immune Regulation in Patients with HIV on Antiretroviral Therapy

**DOI:** 10.1128/mSphere.00798-19

**Published:** 2020-02-05

**Authors:** Medini K. Annavajhala, Sabrina D. Khan, Sean B. Sullivan, Jayesh Shah, Lauren Pass, Karolina Kister, Heather Kunen, Victor Chiang, Gwennaëlle C. Monnot, Christopher L. Ricupero, Rebecca A. Mazur, Peter Gordon, Annemieke de Jong, Sunil Wadhwa, Michael T. Yin, Ryan T. Demmer, Anne-Catrin Uhlemann

**Affiliations:** a Department of Medicine, Division of Infectious Diseases, Columbia University Medical Center, New York, New York, USA; b Microbiome and Pathogen Genomics Core, Columbia University Medical Center, New York, New York, USA; c Department of Orthodontics, School of Dental Medicine, Columbia University Medical Center, New York, New York, USA; d Department of Dermatology, Columbia University Medical Center, New York, New York, USA; e Division of Epidemiology and Community Health, School of Public Health, University of Minnesota, Minneapolis, Minnesota, USA; Icahn School of Medicine at Mount Sinai

**Keywords:** oral microbiome, antiretroviral therapy, mycobiome, immune system activation, HIV, antiretroviral agents, immune dysfunction

## Abstract

Despite evidence of a chronic inflammatory phenotype in people living with HIV (PLWH) on antiretroviral therapy (ART), the role of oral microbiota in chronic immune activation has not been fully explored. We aimed to determine the relationship between oral and gut microbiome diversity and chronic systemic inflammation in ART-treated PLWH with prevalent severe periodontitis, an inflammatory condition commonly associated with HIV infection. We assessed bacterial and fungal communities at oral and gastrointestinal sites in a cohort (*n* = 52) of primarily postmenopausal women on ART using 16S rRNA and internal transcribed spacer (ITS) sequencing and measured cellular and soluble markers of inflammation and immune dysfunction. Linear mixed-effect regression and differential abundance analyses were used to associate clinical characteristics and immunological markers with bacterial and fungal diversity and community composition. Bacterial α-diversity in plaque, saliva, and gut was associated with different immunological markers, while mycobial diversity was not associated with soluble or cellular biomarkers of immune stimulation or T cell dysfunction. Furthermore, lipopolysaccharide-positive (LPS^+^) bacteria previously linked to inflammatory outcomes were enriched at oral sites in patients with severe periodontitis. Fungal α-diversity was reduced in plaque from teeth with higher clinical attachment loss, a marker of periodontitis, and in saliva and plaque from patients with a history of AIDS. Our results show that both bacterial and fungal oral microbiome communities likely play a role in chronic systemic immune activation in PLWH. Thus, interventions targeting both inflammation and the microbiome, particularly in the oral cavity, may be necessary to reduce chronic immune dysregulation in patients with HIV.

**IMPORTANCE** A feedback loop between dysbiotic gut microbiota, increased translocation of microbial products such as lipopolysaccharide, and inflammation has been hypothesized to cause immune system dysfunction in early HIV infection. However, despite evidence of a chronic inflammatory phenotype in patients on antiretroviral therapy (ART), the role of oral microbiota in systemic immune activation and the relationship between oral and gut bacterial and fungal diversity have not been explored. Our study suggests a crucial role for oral bacterial and fungal communities in long-term systemic immune activation in patients on ART, expanding the current paradigm focused on gut bacteria. Our results indicate that interventions targeting both inflammation and microbial diversity are needed to mitigate oral inflammation-related comorbidities, particularly in HIV-positive patients. More broadly, these findings can bolster general models of microbiome-mediated chronic systemic immune activation and aid the development of precise microbiota-targeted interventions to reverse chronic inflammation.

## Abstract (precis)

A feedback loop between dysbiotic gut microbiota, increased translocation of microbial products such as lipopolysaccharide, and inflammation has been hypothesized to cause immune system dysfunction in early HIV infection. However, despite evidence of a chronic inflammatory phenotype in patients on antiretroviral therapy (ART), the role of oral microbiota in systemic immune activation and the relationship between oral and gut bacterial and fungal diversity have not been explored. Our study suggests a crucial role for oral bacterial and fungal communities in long-term systemic immune activation in patients on ART, expanding the current paradigm focused on gut bacteria. Our results indicate that interventions targeting both inflammation and microbial diversity are needed to mitigate oral inflammation-related comorbidities, particularly in HIV-positive patients. More broadly, these findings can bolster general models of microbiome-mediated chronic systemic immune activation and aid the development of precise microbiota-targeted interventions to reverse chronic inflammation.

## INTRODUCTION

Antiretroviral therapy (ART) has allowed people living with human immunodeficiency virus (HIV) (PLWH) to treat HIV infection as a chronic condition due to effective suppression of viremia and restoration of CD4^+^ T cells. Despite ART, however, PLWH have a higher burden of mortality and aging-related inflammatory comorbidity compared to HIV-negative patients (1). In the United States, where more than half of PLWH are over 50 years old (2), these aging-related conditions and their relationship to chronic inflammation are of particular concern.

Early in HIV infection, increased gut permeability (3) potentiates translocation of microbial products such as lipopolysaccharide (LPS) into systemic circulation (4), triggering the release of proinflammatory cytokines (5–7). Due to the link between dysbiosis and microbial translocation (3, 8, 9), a potential causal relationship between immune dysfunction and dysbiotic gut microbiota among PLWH has been proposed (10, 11). Given the increased incidence of gingival inflammation and periodontitis in PLWH (12) and potential deleterious effects of HIV proteins on oral mucosal epithelial tight junctions (13) and keratinocytes (14), HIV infection likely also increases oral microbial translocation. Others have reported associations between oral microbiota and systemic inflammation (15, 16), though the relationship between oral microbiota and immune dysregulation has not been extensively studied in the context of HIV.

Additionally, despite the use of ART, a chronic inflammatory phenotype persists in PLWH (10). Long after infection with HIV, reactivation of latent viral cells in gingival and intestinal mucosa can induce immune cell activation and trigger release of proinflammatory cytokines (17). Antiretroviral agents themselves can increase oral microbial translocation due to inhibition of epithelial cell repair (18) and proliferation (19). Furthermore, immune reconstitution through ART may result in long-term negative impacts on oral T cells and higher expression of inflammatory cytokines in both saliva and gingival crevicular fluid (GCF) (20–25). Targeted study of the interplay between microbial community structure and chronic immune system activation, though, has mainly focused on untreated chronic HIV infection (8, 11, 26, 27), and only recently on PLWH on ART (28). Moreover, the oral mycobiome is emerging as a potentially important factor in periodontitis (29, 30).

Here, we aimed to describe oral and gut microbial and mycobial diversity and community structure in a cohort of PLWH on long-term ART to provide evidence for the hypothesized link between microbial communities and chronic immune dysregulation in patients with HIV.

## RESULTS

### Study cohort.

The study cohort included 52 PLWH on ART (Table 1), with 9 men and 43 women (37 postmenopausal) at a median age of 56 years (interquartile range [IQR], 52 to 61) and consisted of primarily Hispanic (*n* = 29; 56%) and black (*n* = 27; 52%) participants. Follow-up 3 to 6 months after the initial clinic visit was completed for 35 patients (67%). Of the 52 PLWH, 23 (44%) had been diagnosed with HIV for more than 20 years and 16 (31%) had been diagnosed between 10 and 20 years prior to the study. The majority of participants (*n* = 34; 65%) had a documented history of AIDS, defined by a CD4 nadir count of <200 cells/μl and/or a history of opportunistic infections. Almost all study subjects were taking nucleoside/nucleotide reverse transcriptase inhibitors (NRTIs) (93%). Most ART regimens included a combination of NRTIs and an integrase strand transfer inhibitor (INSTI) (29%), a ritonavir-boosted protease inhibitor (PI) (29%), or a nonnucleoside reverse transcriptase inhibitor (NNRTI) (25%). HIV was well-controlled in this population, as expected with long-term ART use. Current CD4 counts were all >200 cells/μl (*n* = 44; 100%), and HIV RNA viral load levels were undetectable (<50 copies/ml) in 98% (*n* = 43) of patients recently tested.

**TABLE 1:** Study cohort characteristics

Characteristic^*a*^	No. of patients (%)^*b*^ (total = 52)
Age, yr [median (IQR)]	56 (52 − 61)
Female	43 (83)
Hispanic	29 (56)
Black	27 (52)
BMI, kg/m^2^ [median (IQR)]	28.8 (25 − 32)
Normal (18 ≤ BMI < 25)	16 (31)
Overweight (25 ≤ BMI < 30)	15 (29)
Obese (30 ≤ BMI ≤ 35)	12 (23)
Morbidly obese (35 ≤ BMI < 45)	8 (15)
Menopausal status	
Premenopausal	5 (10)
Postmenopausal	38 (73)
Hepatitis B	
Prior	8 (15)
Current	7 (13)
Smoker	
Former	5 (10)
Current	11 (21)
Years since HIV diagnosis	
<10 yr	9 (17)
10–20 yr	16 (31)
>20 yr	23 (44)
History of AIDS	34 (65)
CD4 nadir	
<50 cells/μl	10 (19)
<100 cells/μl	4 (8)
<200 cells/μl	16 (31)
ART regimen	
NRTI + PI	15 (29)
NRTI + INSTI	15 (29)
NRTI + NNRTI	13 (25)
Periodontal disease severity^*c*^	
None/mild (≤2 IP sites with CAL > 4 mm)	4 (8)
Moderate (>2 IP sites with CAL > 4 mm)	16 (31)
Severe (>2 IP sites with CAL > 6 mm)	22 (42)
No. of teeth [median (IQR)]	22 (15 − 26)
% teeth with CAL > 4 mm [median (IQR)]	71 (40 − 99)

a Abbreviations: IQR, interquartile range; BMI, body mass index; HIV, human immunodeficiency virus; ART, antiretroviral therapy; NRTIs, nucleoside reverse transcription inhibitors; NNRTI, nonnucleoside reverse transcription inhibitors; PI, protease inhibitors; INSTI, integrase strand transfer inhibitors; IP, interproximal; CAL, clinical attachment loss.

b Number of patients with percentage shown in parentheses unless IQR specified.

c Periodontal disease severity categories as defined by the Centers for Disease Control and Prevention (CDC) and the American Academy of Periodontology (AAP).

### Digestive tract microbiota.

**FIG 1:**
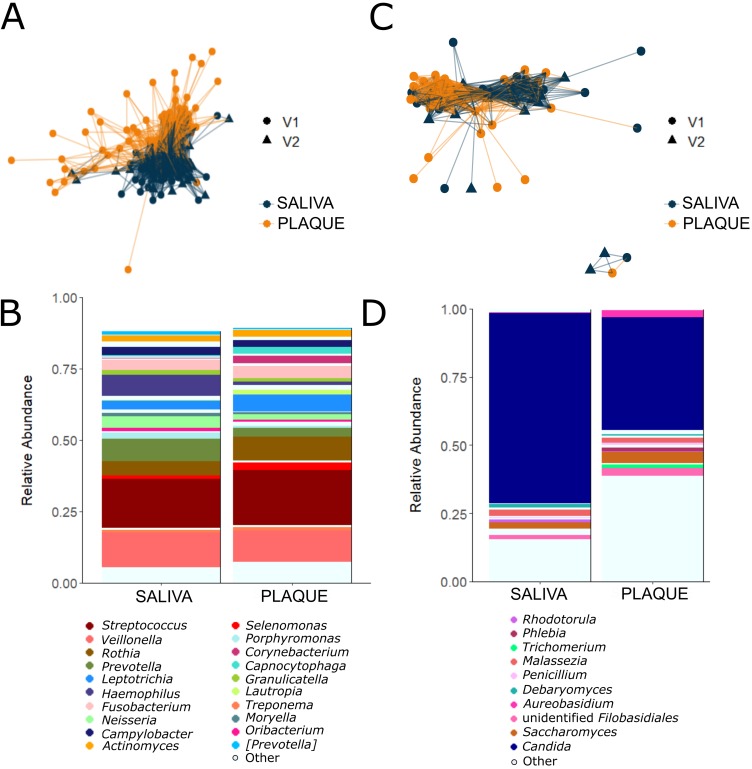
Distinct saliva and subgingival plaque bacterial and fungal communities. (A and C) Bacterial communities differed significantly between saliva (*n* = 84) and subgingival plaque (*n* = 71) samples (A), while fungal communities in saliva (*n* = 43) and plaque (*n* = 42) had greater overlap (C). The thickness of lines reflects pairwise weighted UniFrac distance between samples, and the shapes reflect clinic visit from which the sample was obtained (visit 1 [V1] and visit 2 [V2]). (B and D) Average relative abundance of specific bacterial or fungal genera identified in saliva (*n*_bacterial_ = 84, *n*_fungal_ = 43) and plaque (*n*_bacterial_ = 71, *n*_fungal_ = 42). The top 20 bacterial and 10 fungal genera based on average relative abundance in any sample type are shown in the color legend; all other genera are shown in light blue.

Overall, saliva and plaque samples had distinct bacterial (weighted UniFrac β-diversity index, permutational multivariate analysis of variance [PERMANOVA] *P* = 0.001; Fig. 1A) and fungal communities (weighted UniFrac PERMANOVA *P* = 0.007; Fig. 1C). The average relative abundance of specific bacterial and fungal genera also differed by site (Fig. 1B and D). Both saliva and plaque samples contained relatively high (≥10%) proportions of *Streptococcus* (saliva, median 14.5% [IQR, 9.7 to 21.0%]); plaque, 16.0% [IQR, 11.2 to 22.9%]) and *Veillonella* (saliva, 11.4% [IQR, 7.7 to 16.6%]), plaque, 7.9% [IQR, 3.0 to 17.7%]). Saliva samples contained higher levels of *Prevotella* (7.3% versus 1.9%, Kruskal-Wallis *P* < 0.0001) and *Haemophilus* (5.9% versus 0.07%, Kruskal-Wallis *P* < 0.0001) than plaque samples, while plaque samples contained higher average relative abundance of *Corynebacterium* (1.8% versus 0.0% in saliva, Kruskal-Wallis *P* < 0.0001). Gut microbiome samples contained significant levels of *Bacteroides* (12.7% [IQR, 6.9 to 19.7%]) and *Faecalibacterium* (4.9% [IQR, 2.3 to 10.1%]) as previously reported.

Fungal communities in the saliva were dominated by *Candida* (93.2% [IQR, 48.1 to 98.3%]). Plaque samples, on the other hand, had highly variable levels of *Candida* (7.2% [IQR, 0.0 to 96.6%]), and other lower-abundance genera were also observed, including *Saccharomyces*, *Malassezia*, and *Aureobasidium*.

### Microbial diversity and clinical characteristics.

In the 35 patients who completed follow-up, bacterial Shannon α-diversity tended to be lower at follow-up than at the initial clinic visit in both the saliva (*P* = 0.05) and stool (*P* = 0.08) (see Table S1 in the supplemental material). Therefore, we included clinic visit as an independent variable in subsequent bacterial linear mixed-effect models associating saliva and stool microbial diversity with clinical characteristics of the cohort. Fungal Shannon α-diversity in saliva did not differ by visit (*P* = 0.15), though saliva Chao fungal diversity was significantly lower upon follow-up (*P* = 0.01) (Table S2). Age and race (black versus other/unknown) did not have a significant impact on bacterial α-diversity, though plaque samples from black patients tended to have higher Shannon diversity (*P* = 0.08) (Table S3). Fungal plaque Shannon diversity, on the other hand, was significantly lower in patients who were black compared to all others (*P* = 0.05) (Table S4). Males had higher fungal diversity in saliva (*P* = 0.07; Table S4), though bacterial α-diversity did not differ significantly by sex at any site (Table S3). Menopausal status did not significantly affect bacterial (Table S3) or fungal (Table S4) diversity, although this comparison was likely not adequately powered (five premenopausal women; Tables S1 and S2). Morbidly obese patients had significantly lower saliva Shannon diversity compared to patients with normal body mass index (BMI) (*P* = 0.02; Table S3).

10.1128/mSphere.00798-19.3 TABLE S1 Bacterial α-diversity measures stratified by clinical characteristics. Download Table S1, XLSX file, 0.02 MB. Copyright © 2020 Annavajhala et al. 2020 Annavajhala et al. This content is distributed under the terms of the Creative Commons Attribution 4.0 International license.

10.1128/mSphere.00798-19.4 TABLE S2 Fungal α-diversity measures stratified by clinical characteristics. Download Table S2, XLSX file, 0.01 MB. Copyright © 2020 Annavajhala et al. 2020 Annavajhala et al. This content is distributed under the terms of the Creative Commons Attribution 4.0 International license.

10.1128/mSphere.00798-19.5 TABLE S3 Associations between bacterial Shannon α-diversity and clinical characteristics. Download Table S3, XLSX file, 0.01 MB. Copyright © 2020 Annavajhala et al. 2020 Annavajhala et al. This content is distributed under the terms of the Creative Commons Attribution 4.0 International license.

10.1128/mSphere.00798-19.6 TABLE S4 Associations between fungal Shannon α-diversity and clinical characteristics. Download Table S4, XLSX file, 0.01 MB. Copyright © 2020 Annavajhala et al. 2020 Annavajhala et al. This content is distributed under the terms of the Creative Commons Attribution 4.0 International license.

Bacterial Shannon diversity was higher in patients on NRTI plus NNRTI (NRTI+NNRTI) versus NRTI+PI regimens in both saliva (*P* = 0.05) and plaque (*P* = 0.06) samples (Table S3). In contrast, the use of an ART regimen including an INSTI and NRTI compared to a NRTI+NNRTI regimen, was associated with significantly higher gut diversity (*P* = 0.03). More generally, patients taking INSTIs had higher gut bacterial diversity than those not on INSTIs (*P* = 0.06). Current CD4 levels were above 200 cells/μl in all patients despite different lengths of ART; however, patients with a CD4 nadir below 100 cells/μl tended to have decreased gut bacterial α-diversity compared to those who never had CD4 levels below 200 cells/μl (*P* = 0.08) (Table S3). Current and nadir CD4 values were not significantly associated with bacterial α-diversity at oral sites. Mycobial α-diversity did not significantly differ by ART regimen or by CD4 nadir levels (Table S4). However, we found that subgingival plaque fungal Shannon diversity was significantly lower in patients with a history of AIDS (*P* = 0.02), despite the long-term use of ART.

### Periodontal disease severity and oral microbiota.

Periodontal disease was highly prevalent in this cohort; 74% of patients had at least moderate periodontitis, and 42% had severe periodontitis (Table 1). Clinical attachment loss (CAL) is one of the standard measurements of periodontal disease severity, and it is included in the American Academy of Periodontology (AAP)/Centers for Disease Control and Prevention (CDC) definitions of periodontal disease classifications. CAL, typically reported in millimeters, is indicative of the loss of gingival support at the measured site (tooth) and is calculated as the gingival pocket probing depth plus the distance of gingival recession or minus the distance of gingival overgrowth around the tooth. CAL values of 4 and 6 mm at two or more sites are defined as moderate and severe periodontitis, respectively. The maximum CAL/patient had a median value of 6 mm (IQR, 5 to 7 mm), and the mean CAL/patient was 4 ± 1 mm (± standard deviation). Moreover, we found a large proportion of teeth per individual with CAL ≥ 4 mm (median, 71%; IQR, 40 to 99%) and a high degree of tooth loss (median, 10; IQR, 6 to 17).

Bacterial α-diversity in saliva trended toward higher levels in patients with moderate (*P* = 0.08) and severe (*P* = 0.10) periodontitis compared to those with no or mild disease (Table 2). Bacterial α-diversity in the saliva (Shannon *P* = 0.07; Chao *P* = 0.04) was also inversely associated with the percentage of teeth with CAL ≥ 4 mm, used as a broader marker for periodontal health. Overall bacterial communities (β-diversity) differed significantly with periodontal disease severity as defined by the CDC/AAP in both saliva (weighted UniFrac, PERMANOVA *P* = 0.004) and plaque samples (weighted UniFrac, PERMANOVA *P* = 0.012), with saliva and plaque samples from patients with severe periodontitis clustering separately from those with none/mild periodontitis. Saliva bacterial β-diversity was also significantly associated with the percentage of teeth with CAL ≥ 4 mm.

**TABLE 2:** Associations between bacterial and fungal α-diversity and periodontal disease^*a*^

Organism, sample, and diversity measure^*b*^	Statistic^*c*^	Periodontal disease severity^*d*^			Plaque from teeth with CAL ≥ 4 mm	% teeth with CAL ≥ 4 mm
		None/ mild	Moderate	Severe		
Bacterial						
Saliva						
Shannon	LME *P* value	Ref.	0.08	0.10		0.07
	Coefficient (95% CI)		0.08 (0.00, 0.17)	0.08 (−0.01, 0.16)		0.00 (0.00, 0.00)
Chao	LME *P* value	Ref.	0.18	0.17		0.04
	Coefficient (95% CI)		0.28 (−0.12, 0.67)	0.28 (−0.10, 0.66)		0.00 (0.00, 0.01)
Weighted UniFrac	PERMANOVA *P* value		0.004^*e*^			0.001^*f*^
	*R*^2^		0.070^*e*^			0.482^*f*^
Plaque						
Shannon	LME *P* value	Ref.	0.14	0.32	0.88	0.36
	Coefficient (95% CI)		0.06 (−0.02, 0.14)	0.04 (−0.04, 0.11)	0.00 (−0.05, 0.04)	0.00 (0.00, 0.00)
Chao	LME *P* value	Ref.	0.37	0.78	0.90	0.59
	Coefficient (95% CI)		0.13 (−0.15, 0.40)	0.04 (−0.23, 0.31)	-0.01 (−0.17, 0.15)	0.00 (0.00, 0.00)
Weighted UniFrac	PERMANOVA *P* value		0.012^*e*^		0.561^*f*^	0.019^*f*^
	*R*^2^		0.078^*e*^		0.010^*f*^	0.464^*f*^
Stool						
Shannon	LME *P* value	Ref.	0.34	0.45		0.31
	Coefficient (95% CI)		0.04 (−0.04, 0.12)	0.03 (−0.05, 0.11)		0.00 (0.00, 0.00)
Chao	LME *P* value	Ref.	0.45	0.65		0.46
	Coefficient (95% CI)		0.15 (−0.23, 0.53)	0.09 (−0.28, 0.45)		0.00 (0.00, 0.00)
Weighted UniFrac	PERMANOVA *P* value		0.637^*e*^			0.906^*f*^
	*R*^2^		0.025^*e*^			0.093^*f*^
						
Fungal						
Saliva						
Shannon	LME *P* value	Ref.	0.36	0.88		0.24
	Coefficient (95% CI)		−0.30 (−0.92, 0.32)	−0.05 (−0.64, 0.54)		0.00 (0.00, 0.01)
Chao	LME *P* value	Ref.	0.50	0.72		0.55
	Coefficient (95% CI)		−0.35 (−1.32, 0.62)	−0.17 (−1.10, 0.76)		0.00 (0.00, 0.01)
Weighted UniFrac	PERMANOVA *P* value		0.240^*e*^			0.226^*f*^
	*R*^2^		0.087^*e*^			0.525^*f*^
Plaque						
Shannon	LME *P* value	Ref.	0.69	0.51	0.05	0.22
	Coefficient (95% CI)		−0.12 (−0.69, 0.46)	−0.18 (−0.69, 0.34)	−0.37 (−0.74, −0.01)	0.00 (−0.01, 0.00)
Chao	LME *P* value	Ref.	0.50	0.21	0.02	0.07
	Coefficient (95% CI)		−0.22 (−0.85, 0.40)	−0.38 (−0.94, 0.19)	−0.50 (−0.90, −0.11)	−0.01 (−0.01, 0.00)
Weighted UniFrac	PERMANOVA *P* value		0.179^*e*^		0.287^*f*^	0.150^*f*^
	*R*^2^		0.078^*e*^		0.023^*f*^	0.520^*f*^

a The values show associations between bacterial and fungal diversity and periodontal disease. CAL, clinical attachment loss.

b Shannon and Chao bacterial and fungal α-diversity were log transformed to achieve near-normal distribution (Materials and Methods).

c For linear mixed-effect regression (LME), Shannon (log-transformed) and Chao (log-transformed) α-diversity were considered the outcomes and periodontal disease markers were considered fixed effects in linear mixed-effect models with patient identifier (ID) as a random effect to account for interpatient variability and repeated measures. Clinic visit was included as a potentially confounding fixed effect. PERMANOVA, permutational multivariate analysis of variance.

d Periodontal disease severity scores as defined by CDC/AAP guidelines: severe periodontitis, CAL ≥ 6 mm at ≥2 sites, moderate periodontitis, CAL ≥ 4 mm at ≥2 sites. Ref., reference.

e Overall PERMANOVA *P* and *R*^2^ value based on weighted UniFrac β-diversity by periodontal disease status, adjusted for study identifier (ID) and clinic visit.

f PERMANOVA *P* and *R*^2^ value based on weighted UniFrac β-diversity after adjustment for study ID and clinic visit.

**FIG 2:**
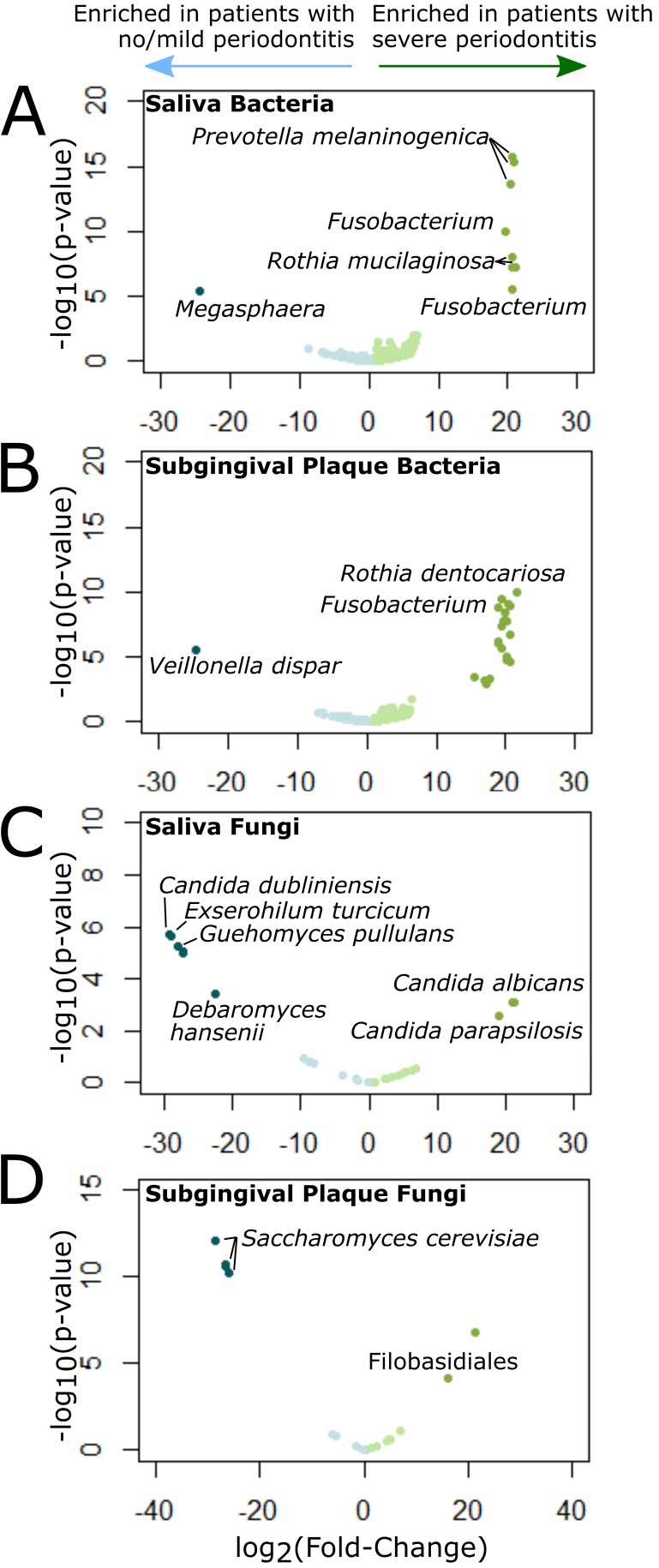
Differentially abundant bacteria and fungi in patients with severe periodontitis. (A to D) Volcano plots showing results of differential abundance analysis comparing bacterial saliva (A) and plaque (B) communities and fungal saliva (C) and plaque (D) communities in patients with severe periodontitis versus no/mild periodontitis as defined by the Centers for Disease Control and Prevention (CDC)/American Academy of Periodontology (AAP). Each volcano plot shows −log_10_ *P* value (vertical axis) and log_2_ fold change (FC) in abundance (horizontal axis) of each operational taxonomic unit (OTU) (represented by points in the plot). Significantly differentially abundant OTUs (*P* < 0.05; FDR < 0.1; | log_2_ FC | > 1) are shown in dark blue (enriched in patients with no/mild periodontitis) or dark green (enriched in patients with severe periodontitis).

Through differential abundance analysis, we found bacterial taxa significantly associated (*P* < 0.05, false-discovery rate [FDR] < 0.05) with periodontal disease severity in saliva and plaque. Patients with severe periodontal disease had saliva with significantly higher levels of Prevotella melaninogenica, Rothia mucilaginosa, and *Fusobacterium*, and plaque with significantly higher levels of Rothia dentocariosa, *Fusobacterium*, *Streptococcus*, and *Prevotella* compared to those with no or mild periodontitis (Fig. 2A and B and Table S5). Patients with CAL ≥ 8 mm at ≥2 interproximal (IP) sites, which we considered a more diseased subset of those with severe periodontitis, also had significantly higher levels of P. melaninogenica and R. mucilaginosa in saliva than those with no or mild periodontal disease, but they did not have higher levels of *Fusobacterium.* Plaques from these patients were enriched in Haemophilus parainfluenzae, *Leptotrichia*, *Lautropia*, Veillonella dispar, *Fusobacterium*, *Catonella*, and several members of the families *Lachnospiraceae* and *Streptococcaceae* and had significantly reduced levels of *Streptococcus*, *Actinomyces*, *Granulicatella*, and *R. dentocariosa* compared to those from patients with no periodontitis.

10.1128/mSphere.00798-19.7 TABLE S5 Differentially abundant bacterial taxa in saliva and subgingival plaque from patients with severe versus no/mild periodontal disease (*DESeq2*, *P* < 0.05, *P*_adj_ [FDR] < 0.05). Download Table S5, XLSX file, 0.01 MB. Copyright © 2020 Annavajhala et al. 2020 Annavajhala et al. This content is distributed under the terms of the Creative Commons Attribution 4.0 International license.

While periodontal disease classification was not associated with changes in mycobial diversity, we found significantly lower fungal α-diversity in subgingival plaque from sites with CAL ≥  4 mm compared to sites with CAL < 4 mm (Shannon *P* = 0.05, Chao *P* = 0.02; Table 2). Fungal α-diversity in the plaque (Chao *P* = 0.07) was also inversely associated with the percentage of teeth with CAL ≥ 4 mm. Saliva samples from patients with severe periodontitis compared to those with no or mild periodontitis were significantly enriched (*P* < 0.05; FDR < 0.05) in Candida albicans and Candida parapsilosis, and had significantly lower levels of Candida dubliniensis, Exserohilum turcicum, Guehomyces pullalans, Debaryomyces hansenii, and three unidentified fungi (Fig. 2C and Table S6). In subgingival plaque, patients with severe periodontal disease had markedly reduced levels of Saccharomyces cerevisiae compared to those with no/mild disease and increased levels of a *Filobasidiales* operational taxonomic unit (OTU) (*P* < 0.05; FDR < 0.05) (Fig. 2D and Table S6).

10.1128/mSphere.00798-19.8 TABLE S6 Differentially abundant fungal taxa in saliva and subgingival plaque from patients with severe versus no/mild periodontal disease (*DESeq2*, *P* < 0.05, *P*_adj_ [FDR] < 0.05). Download Table S6, XLSX file, 0.01 MB. Copyright © 2020 Annavajhala et al. 2020 Annavajhala et al. This content is distributed under the terms of the Creative Commons Attribution 4.0 International license.

### Immunological markers and microbial and mycobial diversity.

**FIG 3:**
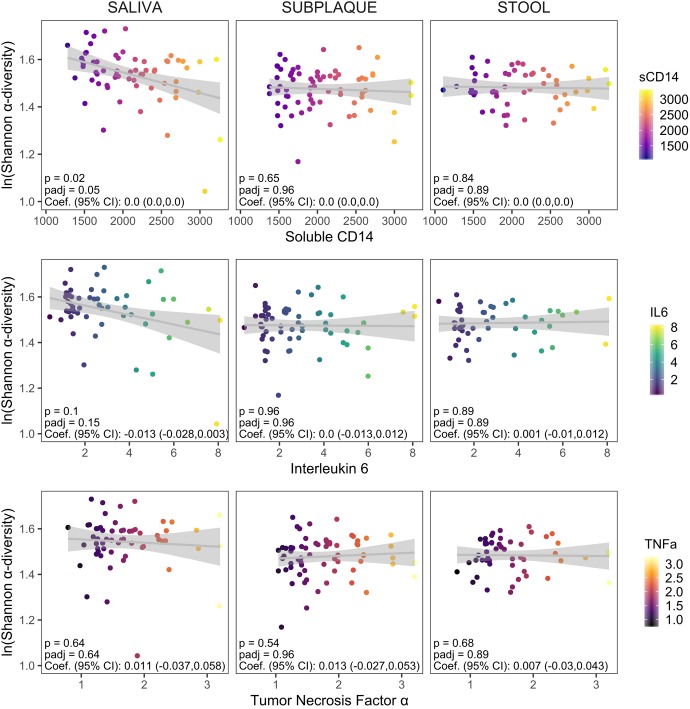
Associations between bacterial α-diversity and serum soluble cytokine levels. Soluble cytokine levels were measured in sera from a majority of the cohort (*n* = 47/52 [90%] from initial clinic visits and *n* = 18/35 [51%] from follow-up visits). We had cytokine levels available for soluble CD14 (sCD14) (*n* = 65), interleukin 6 (IL-6) (*n* = 57), and tumor necrosis factor alpha (TNF-α) (*n* = 61) after excluding samples with values outside the range of detection for each assay. Associations between Shannon α-diversity and levels of serum markers were assessed using linear mixed-effect regression models to account for random effects due to interpatient variability. Unadjusted and FDR-adjusted *P* values (p and padj, respectively) and regression coefficients (Coef.) with 95% confidence intervals (95% CI) are shown in each panel.

We tested for associations between community α-diversity and systemic or cellular markers of inflammation and T cell dysfunction through mixed-effect linear regression, adjusted for repeated measures from initial and follow-up clinic visits (Fig. 3 and Table S7). Bacterial Shannon α-diversity in saliva was inversely associated with soluble CD14 (sCD14) (FDR-adjusted *P* [*P*_adj_] = 0.05; Fig. 3). Differential abundance testing revealed a significant enrichment of *Streptococcus* in the saliva of patients with high sCD14 levels and enrichment of *Neisseria* and R. mucilaginosa in patients with low serum sCD14 (Table S8). An association between interleukin 6 (IL-6) levels and bacterial diversity in saliva was also suggested but not significant (Shannon *P*_adj_ = 0.15). Subgingival plaque and gut diversity, on the other hand, were not associated with any serum cytokine levels.

10.1128/mSphere.00798-19.9 TABLE S7 Associations between bacterial and fungal α-diversity and cellular markers of chronic immune activation and T cell dysfunction quantified from peripheral blood mononuclear cells (PBMCs) derived from mixed-effect linear regression models. Download Table S7, XLSX file, 0.01 MB. Copyright © 2020 Annavajhala et al. 2020 Annavajhala et al. This content is distributed under the terms of the Creative Commons Attribution 4.0 International license.

10.1128/mSphere.00798-19.10 TABLE S8 Differentially abundant bacterial taxa in saliva from patients with high (top tertile) versus low (bottom tertile) serum sCD14 levels (*DESeq2*, *P* < 0.05, *P*_adj_ [FDR] < 0.05). Download Table S8, XLSX file, 0.01 MB. Copyright © 2020 Annavajhala et al. 2020 Annavajhala et al. This content is distributed under the terms of the Creative Commons Attribution 4.0 International license.

In contrast, in evaluating the relationship between α-diversity and cellular markers of systemic inflammation and T cell dysfunction, we found that each sampling site was differently associated with individual cellular markers included in our analysis (Table S7). Saliva α-diversity was associated with CD4/CD8 T cell ratio, a measure of immune dysfunction inversely correlated with morbidity and mortality (31) (Shannon *P*_adj_ = 0.08; Chao *P*_adj_ = 0.03) and with activated CD8 T cells (percentage of HLA-DR^+^ CD38^+^ CD4 T cells, Chao *P*_adj_ = 0.02). Plaque Shannon diversity also trended with the CD4/CD8 T cell ratio (*P*_adj_ = 0.15). Gut Shannon α-diversity, on the other hand, was significantly associated with the percentage of PD1-expressing CD8 T cells, a marker for T cell dysfunction in chronic HIV (Shannon *P*_adj_ = 0.001; Chao *P*_adj_ = 0.002). In all cases, bacterial α-diversity had U-shaped relationships with the levels of these cellular markers, where the middle tertile for CD4/CD8 T cell ratios, activated CD8 T cells, and percentage of PD1-expressing CD8 T cells were associated with changes in microbiome diversity (see Fig. S2 in the supplemental material).

10.1128/mSphere.00798-19.2 FIG S2 Associations between bacterial α-diversity and cellular markers of inflammation and T cell dysfunction. Cellular surface markers were measured by immunostaining and flow cytometric analysis of peripheral blood mononuclear cells (PBMCs) (*n* = 46). Fixable live/dead stain was used to gate out dead cells prior to further gating of CD3^+^ T cells. HLA-DR^+^ CD38^+^ double-positive T cells were used as markers of chronic immune activation, whereas T cell exhaustion/dysfunction was measured using the percentage of CD8^+^ and CD4^+^ T cells expressing PD-1. Fixed samples (4% PFA) were measured on the LSRII and analyzed using FlowJo software. Associations between Shannon index α-diversity and cellular markers were assessed using linear mixed-effect regression models to account for random effects due to interpatient variability. Unadjusted and FDR-adjusted *P* values and regression coefficients with 95% confidence intervals are provided in Table S7 in the supplemental material. Download FIG S2, TIF file, 0.5 MB. Copyright © 2020 Annavajhala et al. 2020 Annavajhala et al. This content is distributed under the terms of the Creative Commons Attribution 4.0 International license.

Fungal Chao diversity in saliva inversely trended with soluble CD14 levels (*P*_adj_ = 0.15), although neither saliva nor plaque fungal Shannon diversity was associated with serum cytokine levels. Fungal α-diversity in saliva trended with the levels of activated CD4 T cells (percentage of HLA-DR^+^ CD38^+^ CD4, Shannon *P*_adj_ 0.1 and Chao *P*_adj_ = 0.07). The α-diversity of fungal communities in plaque was not associated with any cellular markers tested.

## DISCUSSION

Our results revealed distinct microbial and mycobial communities along the digestive tract of PLWH on ART. While a recent study reported significantly higher oral bacterial diversity after 24 weeks on ART in participants with low CD4 counts (32), patients in our cohort with a CD4 nadir below 100 cells/μl had lower gut bacterial diversity, and patients with a history of AIDS had significantly lower subgingival plaque fungal α-diversity, despite more than 75% of the cohort having been diagnosed and treated with ART for over 10 years.

Furthermore, we found that the use of specific ART regimens correlated with alterations in both gut and oral bacterial diversity, though differently at each site. The use of protease inhibitors with NRTIs was associated with decreased oral diversity (saliva and plaque) compared to an NRTI+NNRTI regimen. In the gut, on the other hand, an INSTI combined with NRTIs showed significantly higher α-diversity compared to a combination of NRTI and NNRTI. The directionality of the relationship between HIV infection, development of AIDS, and ART regimen and bacterial diversity could not be determined within this study design. However, these results imply long-term effects of HIV infection on the mycobiome in addition to the bacterial microbiome, which are either not completely mitigated by ART or represent effects of the treatment itself.

Our cohort of primarily postmenopausal women with HIV had a remarkably high prevalence of severe periodontal disease and poor overall periodontal health, despite treatment with ART. Comparison of this cohort to published data from the 2009–2012 National Health and Nutrition Examination Surveys (NHANES) suggested a much higher burden of severe periodontal disease, 64% in our cohort compared to 6.4% in postmenopausal women without HIV (33). Furthermore, bacterial α- and β-diversity in saliva differed significantly with increasing periodontal disease severity. We also observed significantly lower fungal α-diversity in subgingival plaque from teeth with severe loss of tissue attachment (CAL ≥ 4 mm). Additionally, several bacterial and fungal taxa were significantly enriched in patients with severe periodontitis. Of note, most of these taxa correspond to Gram-negative organisms known to harbor LPS, which has been implicated in immune system activation (34). Furthermore, the high prevalence of severe periodontitis allowed for differential abundance comparisons between severe and no periodontal disease, unlike prior reports which largely compared healthy patients to those with any level of periodontitis (35). Thus, our analysis revealed the enrichment in patients with severe periodontitis of organisms not consistently associated with disease in previous reports (e.g., R. mucilaginosa, P. melaninogenica) (35, 36).

Saliva, subgingival plaque, and gut microbiota were differently associated with levels of soluble and cellular markers of inflammation and T cell dysfunction. Notably, the absence of association between plaque and especially gut microbiota and soluble cytokine markers was unexpected. More importantly, we found that oral microbial diversity correlated with multiple cellular immune markers. Although the current literature largely assumes that gut dysbiosis is most critical in the development of an inflammatory phenotype, our results imply that oral microbiota play an equally, if not more, important role in chronic immune activation. This highlights the importance of studying microbial communities in various sites along the digestive tract for a more comprehensive understanding of dysbiotic states and their impact on immune system activation. A prior study found elevated immune markers in patients with HIV versus uninfected controls, yet no significant correlation between the oral microbiome and immune response (37). However, the patients with HIV were primarily older homosexual men rather than postmenopausal women. Also, patients with fewer than 20 teeth were excluded, resulting in a lower burden of severe periodontitis than in our study.

Microbial translocation secondary to dysfunctional barrier immunity has been hypothesized to rely heavily on the early development of microbial dysbiosis (3, 8, 9). Thus, the selection for “inflammophilic” taxa in the hyperinflammatory environment of early HIV (38) may result in allogeneic succession of the digestive tract microbiome toward dysbiosis, and ultimately increase microbial translocation. Our results suggest that digestive tract diversity is associated with varied outcomes long after initiation of ART, possibly enabling an ongoing bidirectional feedback loop of persistent microbiome signatures, microbial translocation, and immune system activation. Gut mucosal barrier dysfunction and persistent immune activation had been shown previously in patients with HIV (26, 28, 39). However, here we demonstrate that oral taxa are also correlated with systemic immune response. We found that decreased diversity of plaque and saliva microbiota was associated with history and treatment of HIV, periodontal disease severity, and immunological markers. Future studies incorporating uninfected controls are necessary to further inform this notion.

Several limitations to our study need to be considered. This was a single center study that mainly included postmenopausal women of Hispanic ethnicity, which limits the generalizability of our findings. Our sample size was small, and we may have lacked power for some comparisons, especially with respect to sex and menopausal status. Also, due to loss in follow-up, paired biospecimens were not available for all participants, limiting sample size for longitudinal analyses. Nevertheless, our study provides important data on the potential roles of oral bacterial and fungal microbiomes, immune dysfunction, and periodontal disease in patients on ART up to decades after initiation of treatment. Moreover, we expand the current literature regarding chronic inflammation and microbial dysbiosis in patients with HIV to suggest a crucial role for persistent oral microbial markers in long-term systemic immune activation. The establishment of the bidirectional feedback loop described above prior to clinical presentation of inflammatory diseases, such as periodontitis, suggests that characterization of preclinical microbial dysbiosis may improve the chances of early prevention. Furthermore, our results point to the need for interventions targeting both inflammation and dysbiosis in order to mitigate the high morbidity and mortality of PLWH, in particular for oral inflammation-related comorbidities. More broadly, these results are applicable to general models of microbiome-mediated chronic systemic immune activation and the development of precise microbiota-targeted interventions and therapeutics to reverse chronic inflammation.

## MATERIALS AND METHODS

### Study population and sample collection and processing.

This study was approved by the Columbia University Institutional Review Board. Written informed consent was obtained from all study subjects. We recruited patients between September 2016 and September 2017 from the HIV Outpatient Comprehensive Health Program at Columbia University Irving Medical Center and ongoing studies of bone health in postmenopausal women (40–45). Inclusion criteria were HIV-positive (HIV+) diagnosis with ≥6 months of ART. Exclusion criteria included age of <18 years, current chemo- or immunotherapy, or antibiotic use in the preceding 3 months other than prophylactic medications for opportunistic infections. We reviewed electronic medical records to obtain sociodemographic information and dental and medical histories.

Patients first provided ≥5 ml saliva, which was stored at –80°C as both whole saliva aliquots and biofilm resuspended with Mo Bio PowerSoil PowerBead solution in garnet bead tubes (Qiagen). Patients underwent dental examination and subgingival plaque collection using a single scaling stroke at the gingival pocket base from the distal site of six index teeth based on the Human Microbiome Project (46) (two molar teeth [teeth number 3 and 19], two premolar [teeth 12 and 28], and two incisor teeth [teeth 9 and 25] or alternative teeth in the same quadrant). Plaques were stored in Tris-EDTA (TE) buffer at –80°C within 2 h. Periodontal disease severity was defined according to the Centers for Disease Control and Prevention (CDC)/American Academy of Periodontology (AAP) based on clinical attachment loss (CAL) (Table 1). For a broader marker for periodontal health, we calculated the percentage of teeth with CAL of ≥4 mm. Plaque was also categorized as obtained from “nonsevere” or “severe” teeth based on CAL at collection site (< or ≥4 mm CAL, respectively).

Patients were asked to provide stool samples within 24 h of enrollment, which were aliquoted and stored at –80°C. Ficoll density centrifugation was used to separate blood samples into peripheral blood mononuclear cells (PBMC), which were separated into ≥5 × 10^6^ cell aliquots and stored at –80°C in 10% dimethyl sulfoxide (DMSO) and 90% fetal bovine serum (FBS) for immunostaining, and serum samples which were stored at –80°C for cytokine testing. Saliva, stool, and blood samples were again collected at a second clinic visit after 3 to 6 months.

### DNA extraction, library preparation, and microbial sequencing. (i) Sample selection and categorization for sequencing.

We performed 16S rRNA sequencing on all available saliva (*n* = 52) and stool (*n* = 46) samples collected from initial clinic visits and all 35 saliva and 30 stool samples available from follow-up visits (see Fig. S1 in the supplemental material). Plaque samples were paired for each patient based on the highest and lowest CAL score of the collected subgingival plaques. We sequenced 84 plaque samples, including paired plaque samples from 40 patients and 1 unpaired plaque from each of 4 patients with no additional plaques available. The remaining eight patients refused plaque collection, were missing teeth at the specified sampling sites, or were fully edentulous. Due to unreliable fungal amplification from stool samples, potentially linked to a recent report suggesting a lack of routine gut mycobiome colonization (47), only saliva and plaque samples were included in the mycobial analysis. We selected 43 saliva and 42 plaque samples (unpaired samples all from diseased teeth) for internal transcribed spacer (ITS) (fungal) sequencing.

10.1128/mSphere.00798-19.1 FIG S1 Study flowchart. Our study included 52 patients with HIV on ART, 35 of whom completed follow-up within 6 months. We collected saliva, stool, and blood samples from initial clinic visits (saliva, *n* = 52; stool, *n* = 46; blood, *n* = 47) and follow-up visits (saliva, *n* = 35; stool, *n* = 30; blood, *n* = 29). We collected a total of 233 subgingival plaques from 44 patients (median 3 plaques per patient). The remaining eight patients refused plaque collection, were missing teeth at the specified sampling sites, or were fully edentulous. Download FIG S1, TIF file, 1.3 MB. Copyright © 2020 Annavajhala et al. 2020 Annavajhala et al. This content is distributed under the terms of the Creative Commons Attribution 4.0 International license.

### (ii) DNA extraction.

DNA was extracted from saliva using the Mo Bio Ultraclean kit (Qiagen) and from dental plaque using the MasterPure Gram-Positive Purification kit with an 18-h incubation. DNA from stool samples was extracted using the MagAttract PowerSoil DNA kit on an Eppendorf epMotion 5075 Liquid Handling Workstation.

**(iii) Library preparation and sequencing.** For bacterial sequencing, we amplified the V3-V4 regions of the 16S rRNA gene using standard primers (48) with Illumina Nextera adaptors (Illumina). We profiled fungal communities using the ITS1 region (49), amplified using published primers (forward primer ITS1F and reverse primer ITS2R) (50) with adaptors for Illumina multiplexing and an adapted cycling protocol (51). PCR products were purified using Agencourt AMPure XP beads (Beckman Coulter) and multiplexed using the Illumina Nextera XT Index kit. Indexed 16S rRNA and ITS libraries were purified using AMPure XP beads and quantified using the Quant-iT Broad Range dsDNA assay kit (Thermo Fisher Scientific). Libraries were normalized and pooled with a 10% PhiX spike and sequenced on an Illumina MiSeq with a v3 kit. Negative controls were included on all sequencing runs, and a Candida glabrata positive control was included in ITS amplification and sequencing.

### 16S rRNA and ITS microbiome analysis.

16S rRNA and ITS sequences were processed using Quantitative Insights Into Microbial Ecology 2 (QIIME2) (52) and R v3.3.0 (53). Demultiplexed FASTQ sequences were quality filtered, trimmed, dereplicated, and filtered for chimeric sequences using pair-ended DADA2 (54), resulting in exact sequence variant (feature) tables. Features with an average relative abundance of <0.0005% across all samples were filtered using *phyloseq* v1.19.1 (55). Filtered features were used to create phylogenetic trees using MAFFT and FastTree in QIIME2. The QIIME2 Naïve Bayes taxonomic classifier was trained on the Greengenes (56) 97% operational taxonomic unit (OTU) clustered database for 16S feature classification, and the UNITE 97% database (57) and an ITS1-specific ntF database (58) for ITS feature classification. *phyloseq* was used to calculate α-diversity (Shannon, Chao) and β-diversity (weighted UniFrac). On the basis of α-diversity rarefaction, we selected appropriate minimum feature count cutoffs of 5,000 (16S) and 3,000 (ITS) for analysis. These cutoffs resulted in 84 saliva, 72 stool, and 71 plaque samples available for bacterial analysis, including 34 high-low CAL paired plaques and 3 unpaired plaques (Fig. S1). All 43 saliva and 42 plaque fungal samples had sufficient counts. Negative controls produced read counts well below the minimum cutoffs used (50 to 320 reads).

### Measurement of cytokines and inflammatory cellular markers.

Cytokine levels and monocyte activation were measured using commercial enzyme-linked immunosorbent assays (ELISAs) (proinflammatory: interleukin 6 [IL-6], tumor necrosis factor alpha [TNF-α]; monocyte activation: soluble CD14 [sCD14]) in sera from a majority of the cohort (*n* = 47/52 [90%] from initial clinic visit and *n* = 18/35 [51%] from follow-up visit). Human Quantikine Immunoassay controls were included on all ELISAs (R&D Systems). Serum samples were diluted 200-fold for sCD14 assays according to the manufacturer’s instructions and undiluted for IL-6 and TNF-α assays. Of the 65 available serum samples, we were able to analyze biomarker data available for sCD14 (*n* = 65), IL-6 (*n* = 57), and TNF-α (*n* = 61) after excluding samples with serum marker levels outside the range of detection for each assay.

Cellular surface markers were measured by immunostaining and flow cytometric analysis of PBMC (*n* = 46). Fixable live/dead stain was used to gate out dead cells prior to further gating of CD3^+^ T cells. HLA-DR^+^ CD38^+^ double-positive T cells were used as markers of chronic immune activation, whereas T-cell exhaustion/dysfunction was measured using the percentage of CD8^+^ and CD4^+^ T cells expressing PD-1. Fixed samples (4% paraformaldehyde [PFA]) were measured on the LSRII instrument and analyzed using FlowJo software.

### Statistical analysis.

Samples from initial and follow-up visits were included in linear mixed-effect regression analyses to account for within-patient correlation and clinic visit where applicable (i.e., for saliva and stool samples, collected at both visits, but not for plaque, which was collected only during the initial visit). The effects of patient clinical history variables, serum cytokine levels, and inflammatory cellular markers (fixed effects) on richness and evenness (Shannon and Chao α-diversity, outcome) of saliva, plaque, and stool microbial communities were assessed by linear mixed-effect analysis using *lme4* and *lmerTest*, with intercepts for study subjects included as random effects. We used the Benjamini-Hochberg FDR method to adjust *P* values from associations of soluble and cellular markers with microbiome diversity to account for multiple testing. Bacterial and fungal Shannon and Chao α-diversity values were log transformed (ln) to achieve a normal-like distribution, as assessed using QQ-plot visualizations. Principal-coordinate analysis (PCoA) using *phyloseq* and permutational multivariate analysis of variance (PERMANOVA) using *vegan* (59) were used to test differences in microbial community composition (β-diversity) across groups based on pairwise weighted UniFrac distances. *DESeq2* (60) was used to identify differentially abundant bacterial taxa after multiple-comparison *P* value adjustment.

### Data availability.

All 16S and ITS FASTQ sequence files have been deposited in the NCBI Sequencing Read Archive (SRA) under BioProject accession number PRJNA471556 and SRA study accession number SRP146270.
