# Supplementary figures and images for: Oral and Gut Microbial Diversity and Immune Regulation in Patients with HIV on Antiretroviral Therapy
Source: mSphere. 2020 Feb 5;5(1):e00798-19. doi: 10.1128/mSphere.00798-19 (PMC7002309; doi:10.1128/mSphere.00798-19)

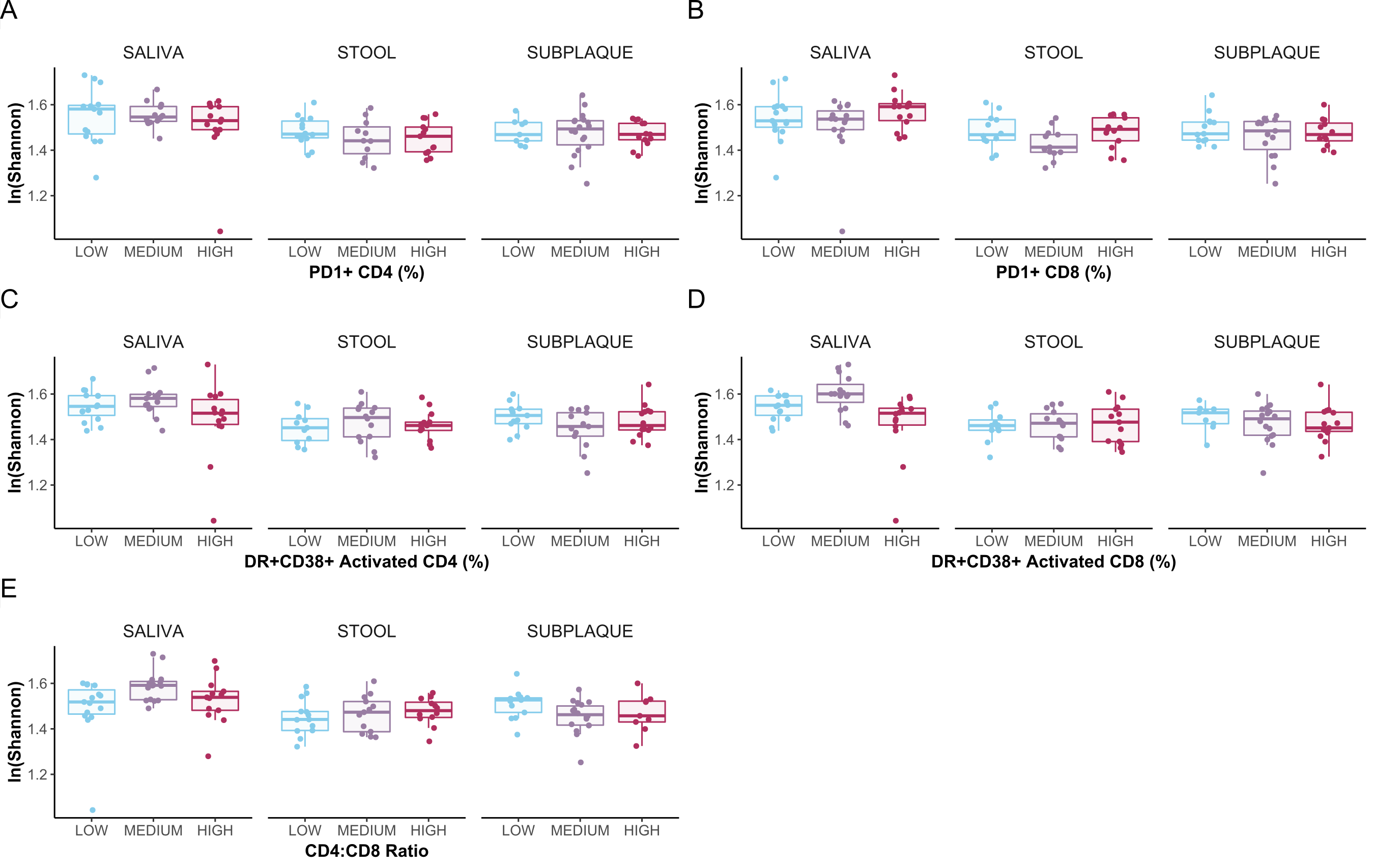

Supplement: FIG S2 [file mSphere.00798-19-sf002.tif]

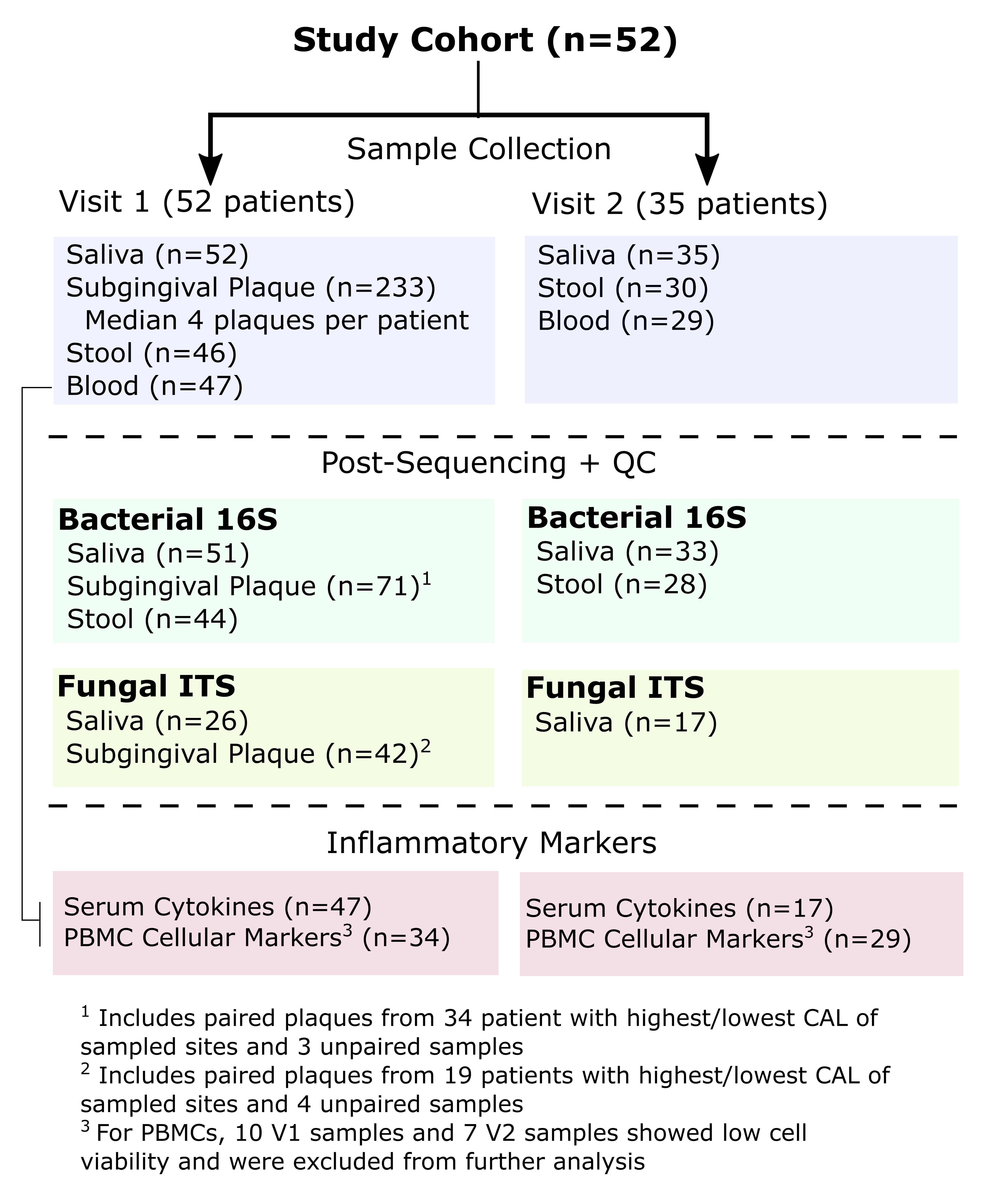

Supplement: FIG S1 [file mSphere.00798-19-sf001.tif]
